# Opportunities and challenges of implementing an urban primary healthcare delivery model: Programmatic lessons from Aalo Clinic, Bangladesh

**DOI:** 10.1371/journal.pone.0341924

**Published:** 2026-02-09

**Authors:** Khadija Islam Tisha, Mohammad Wahid Ahmed, A M Rumayan Hasan, Orin Akter, Md. Golam Rabbani, Chandrasegarar Soloman, Margub Aref Jahangir, Maya Vandenent, Shehrin Shaila Mahmood

**Affiliations:** 1 Health Systems and Population Studies Division, icddr, b, Dhaka, Bangladesh; 2 UNICEF Bangladesh, Dhaka, Bangladesh; World Health Organization, Regional Office for South-East Asia, INDIA

## Abstract

**Background:**

Rapid urbanization in Bangladesh has strained the healthcare system, leaving urbanites underserved in primary healthcare (PHC). To address this gap, a UNICEF-supported Government of Bangladesh initiative introduced the Aalo Clinic model to expand access to affordable PHC. This study explored implementation challenges and opportunities of the Aalo Clinic model from a programmatic perspective for strengthening urban PHC delivery.

**Methods:**

The opportunities and challenges of implementation were explored through qualitative research conducted between June 2022 and May 2023 in six Aalo Clinic intervention areas across four city corporations in Dhaka, Bangladesh. Twenty-four in-depth interviews with service providers and eighteen key informant interviews with clinic-in-charges, community leaders, scheme operators, and policymakers were conducted, along with periodic observations at clinics. A thematic analysis was performed to analyze the data.

**Results:**

Three core themes evolved from the analysis – technical, administrative, and financial opportunities and challenges. Aalo Clinic’s integrated digitalized health platform reported to streamline operations through medical documentation and real-time monitoring. Initiatives like prescription audits and client feedback systems reported to promote rational prescribing and delivering quality care. However, key challenges included limited choice of medicine, interrupted internet connection, high patient flow, and longer waiting times. Despite challenges policymakers mentioned Aalo Clinic’s potential to integrate into the national health system, highlighting the need for coordinated efforts to overcome obstacles. Financial sustainability, however, hinges on the model’s integration into the government system and the provision of public funding to support its implementation.

**Conclusion:**

The study underscores the importance of addressing implementation challenges while capitalizing on opportunities to scale up the Aalo Clinic model. Recommendations include implementing structured monitoring, ensuring need-based medicine supply, fostering collaboration with the government for referrals, and facilitating cross-learning among urban PHC facilities. By addressing implementation challenges and strengthening multisectoral collaboration, the Aalo Clinic model shows potential to contribute to improved access to quality PHC in urban settings, in line with the broader vision of health for all.

## Introduction

Urbanization poses challenges in developing nations like Bangladesh, with rapid expansion straining the healthcare system [1]. Bangladesh is experiencing rapid urbanization, with 44% of the country’s population living in urban areas in 2021 compared to 24% in 2001 [2]. This surge in urbanization, coupled with a constant influx of internal migrants and the proliferation of slums in major cities, places continuous pressure on the urban health system [3]. Despite socioeconomic advancements, including poverty reduction, increased women’s education, and enhanced life expectancy [4,5], disparities in healthcare accessibility persist between rural and urban areas. In urban settings, these gaps are largely attributed to a due to a weak and fragmented urban health system [6].

In Bangladesh’s pluralistic health system, primary healthcare (PHC) is the first level of contact between individuals and the health system. PHC includes promotive, preventive, and curative services. Core PHC services include maternal, newborn, and child health services, reproductive health and family planning services, health education and counselling, and management of minor ailments [4]. In urban Bangladesh, divided responsibilities between the Ministry of Health and Family Welfare (MoHFW) and the Ministry of Local Government, Rural Development and Cooperatives (MoLGRDC) contribute to a less coherent PHC system compared to rural [7]. Currently, the MoLGRDC offers PHC through the Urban Primary Healthcare Services Delivery Project (UPHCSDP) in partnership with non-government organizations (NGO) and government outdoor dispensaries (GOD), which are inadequate in number and falls short of meeting the needs of growing urban population and remain heavily dependent on development partner’s budget [5].

Service coverage, one of the key Sustainable Development Goals (SDG) indicators for Universal Health Coverage (UHC), is limited by a fragmented urban health system, weak accountability, and limited access to PHC. Another key indicator, catastrophic healthcare expenditure, stems from inadequate public investment in health and the absence of financial protection schemes. With a large informal sector in Bangladesh, achieving financial protection for health relies on expanding public financing. The urban poor are disproportionately affected by a weak health system, leading to experiencing low-quality care, using unregulated private sector while incurring out-of-pocket expenditure (OOPE) due to absence of accessible and quality public PHC services [8,9]. In absence of accessible PHC, patients often seek care from informal providers [10], which may contribute to delays in appropriate treatment.

Strengthening PHC is the most cost-effective strategy in low- and middle-income countries (LMICs) to achieve UHC [11,12]. Despite this emphasis on PHC, its performance remains inadequate in LMICs, necessitating substantial improvement to align with the SDGs and attain UHC [13]. In response to these challenges, several PHC models have been implemented in LMICs. One notable example is Delhi’s Mohalla Clinic, which effectively reaches urban populations through a community-based approach, particularly benefiting the poor [14]. Research indicates that these clinics significantly improved healthcare access for the urban poor, reducing time and costs for availing treatment [15]. This increased access encouraged early treatment-seeking, consequently alleviating the burden on higher-level healthcare facilities [16]. In Bangladesh, private and NGO sectors are also embracing innovative models to enhance preventive and clinical care provision by delivering doorstep and mobile health services to the urban poor, slum dwellers, and factory and office workers.

Despite growing evidence on PHC models from LMICs [17–19], there remains limited evidence on the implementation experiences of innovative urban PHC models specifically tailored to the rapidly expanding urban populations in Bangladesh. Understanding how such models function in real-world settings is crucial for informing urban health reforms and achieving UHC targets. Recently in Bangladesh, recognizing the crucial role of PHC, a UNICEF-supported Government of Bangladesh program introduced a comprehensive urban PHC model named ‘Aalo Clinic’. The Aalo Clinic model aims at providing accessible, affordable care to urbanites, particularly low-income populations. It provides a comprehensive range of PHC services in morning and evening shifts through a digitalized platform, offering a variety of medicines and diagnostic tests free of cost [20]. This study aimed to explore the implementation challenges and opportunities of the Aalo Clinic model from a programmatic perspective in improving urban PHC services in Bangladesh. Strengthening evidence on urban PHC models, such as Aalo Clinic, is critical for informing policy in Bangladesh, where rapid urbanization continues to exacerbate health inequities. Findings from this study can guide policymakers, and development partners to scale up or refine PHC models, improving access, quality, and equity of PHC for urban populations in Bangladesh and similar LMIC settings.

## Methods

### Study design

This paper reports on the qualitative component of a broader implementation research study conducted from 1 June 2022 to31 May 2023. A qualitative descriptive design was applied, using in-depth interviews (IDIs), key informant interviews (KIIs), and facility observations to generate a comprehensive understanding of implementation experiences.

### Study settings

The study was conducted during the pilot phase of the Aalo Clinic model in six intervention areas located across four City Corporations in Bangladesh. Study sites included Korail and Mirpur slums (Dhaka North City Corporation); Shyampur, and Dhalpur slums (Dhaka South City Corporation); Tongi-Ershad Nagar slum (Gazipur City Corporation) and Narayanganj low-income area (Narayanganj City Corporation). These sites were purposively selected as the Aalo Clinic model was implemented in these locations. These locations were purposively selected as part of the Aalo Clinic’s implementation strategy, which prioritized proximity to slum settlements to ensure accessibility for the urban poor, while maintaining a universal service delivery approach for patients from all socioeconomic backgrounds. Each clinic operates six days a week in two-shifts (morning shift from 8:30 am to 2:30 pm and an evening shift from 3:00 pm to 9:00 pm) and is staffed to maintain gender equity and comprehensive service provision. During every shift, one male and one female physician provide consultations, while one paramedic, one nurse, and an additional lab technologist support service delivery. Clinics are designed to facilitate integrated PHC services within the community, with consultation rooms, a laboratory section, a pharmacy, and a waiting area.

### Study participants and sampling

Purposive sampling was used to select participants with direct involvement in Aalo clinic operations and service delivery. A total of 42 interviews were conducted, comprising 24 IDIs with service providers (including physicians, nurses, paramedics, lab technicians, lab managers, and clinic assistants) and 18 KIIs with clinic in-charges, scheme operators (program personnel responsible for managing operations, coordinating with providers, and overseeing supply chains and monitoring), facility managers, community leaders, and policymakers. Participants were selected based on the following criteria: a) Individuals directly involved in Aalo Clinic service delivery, including physicians, nurses, paramedics, lab technicians, lab managers, and clinic assistants; b) Individuals involved in program implementation or monitoring, including clinic in-charges, scheme operators, and facility manager; and c) Individuals with relevant knowledge or engagement in Aalo Clinic operations, including community leaders and policymakers. Participants who met these criteria but were unwilling to participate were excluded. The diversity of participant roles ensured that a wide range of operational perspectives were captured. In addition, non-participant facility observations were conducted across all six Aalo clinics to contextualize and triangulate interview findings. Each facility was visited on three separate occasions during the study period covering both morning and evening service shifts, resulting in a total of 18 observation visits. Observations focused on clinic infrastructure, staffing, workflow, service processes, and patient flow dynamics. Field notes from these visits were used to complement and verify themes emerging from interviews, helping to identify consistencies or discrepancies in provider-reported experiences and observed practices.

### Data collection

Data were collected by experienced qualitative researchers using semi-structured interview guides developed based on literature and study objectives (included in S1 Appendix). Interviews were conducted face-to-face at locations convenient for participants, ensuring privacy, comfort, and confidentiality. Each interview lasted 45–60 minutes and was audio-recorded with participants’ consent. Field notes were taken during interviews to capture contextual information and non-verbal cues. Facility observations were conducted using structured checklists combined with open-ended field notes to document operational processes and facility readiness.

### Data analysis

All interviews were audio-recorded, transcribed verbatim in Bengali, and de-identified for analysis. To ensure accuracy and quality, the researchers who conducted the interviews cross-checked all transcripts. Thematic analysis was carried out by following Braun and Clarke’s 6-steps thematic approach [21]. Once the data was verified, the research team began familiarizing themselves with the transcripts through repeated reading. The analysis team familiarized themselves with the data by repeatedly and thoroughly reading transcripts from a subset of group discussions. A coding framework was developed combining deductive codes derived from the study objectives and literature on PHC implementation in LMICs, along with inductive codes emerging directly from the data. Initially, two researchers independently applied codes to the data. To ensure consistency in code application, the team convened regularly to review and deliberate on the coding, addressing both agreements and discrepancies. The codes were then clustered into thematic categories that highlighted opportunities and challenges of implementing Aalo Clinic model from programmatic perspectives. A thematic analysis matrix was developed based on the themes, using Microsoft Excel. After that, the themes were reviewed and revisited, and labelled and re-labelled. Finally, the illustrative quotes were chosen, and the findings were presented and contextualized. The overall analysis was supervised by the senior researchers. To ensure credibility and dependability, triangulation among different researchers was maintained throughout the analysis. Field notes and observational data were integrated with interview transcripts to facilitate triangulation during analysis. Additionally, peer debriefing and daily debriefing throughout fieldwork enhanced the rigor of the analysis. Due to resource constraints, member checking was not conducted. The process continued until thematic saturation was reached, whereby no new codes or themes emerged from the data. During analysis and reporting, illustrative quotations were translated into English and reviewed by the research team to ensure accuracy and preservation of meaning. Facility observation data were analyzed alongside interview data to provide contextual understanding and enrich emerging themes.

### Ethical consideration

This study was approved by the Institutional Review Board of the International Centre for Diarrhoeal Disease Research, Bangladesh (icddr,b) (Protocol number: PR- 22020). Informed written consent was obtained from all the participants prior to the interviews, and confidentiality, and anonymity were ensured at every stage of the study. All identifiable information, including phone numbers and unique participant IDs, was collected and stored on password-protected devices and encrypted servers accessible only to authorized research personnel. During data linkage, unique IDs were used to anonymize personal identifiers, minimizing the risk of re-identification.

## Results

Table 1 presents the characteristics of the 42 study participants, comprising 24 IDIs and 18 KIIs. In terms of gender distribution, the majority of IDI participants were female (16 of 24), whereas most KII participants were male (14 of 18). Most IDI participants were aged 20–29 years, while the majority of KII participants were aged 40 years and above.

**Table 1:** Characteristics of study participants (n = 42).

	In-depth interviews n = 24	Key informant interviews n = 18
**Designation**		
Physician	5	**–**
Nurse	6	**–**
Paramedic	5	**–**
Lab technician	4	**–**
Lab manager	1	**–**
Clinic assistant	3	**–**
Clinic in-charge	**–**	3
Scheme operator	**–**	5
Facility manager	**–**	1
Community leader	**–**	4
Policymaker	**–**	5
**Gender**		
Male	8	14
Female	16	4
**Age category**		
20-24	7	**–**
25-29	5	**–**
30-34	4	**–**
35-39	4	3
40-44	2	5
45+	2	10

**Fig 1:**
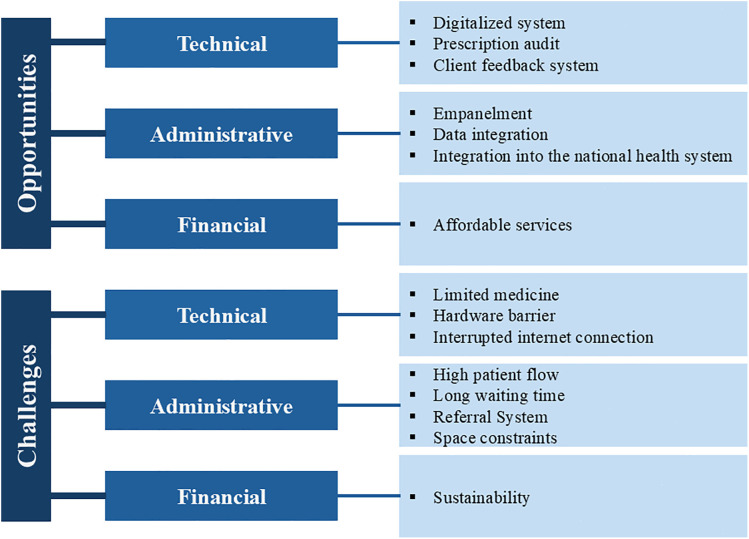
Thematic summary of implementation opportunities and challenges of the Aalo Clinic model.

Three core themes on the implementation-related opportunities and challenges evolved from the analysis– i) technical, ii) administrative, and iii) financial. Each theme is further grouped into sub-themes (Fig 1).

### Opportunities

#### Technical.

***Digitalized system*:** The Aalo Clinic has been operated on the Integrated Digitalized Health Platform (IDHP), a digital system. IDHP was reported by participants to be used for patient registration, medical documentation, and inventory management. Upon initial visit, each patient received a unique identification number. Patients' medical history, prescriptions, and diagnostic reports were recorded in the digital health information system, allowing physicians to access these records during follow-ups using the unique ID or patients' registered phone number. This system was described as easing patients’ burden of carrying around medical documents or worrying about losing them.

*“(IDHP) assign a unique ID. All data entries are linked to this ID and patients' mobile phone number. I can access prescriptions and lab reports directly through the system. Patients don’t need to carry their documents around.”* (IDI_03_Clinic assistant)

Additionally, service providers reported that the digitalized system reduced staff workload and saved time by replacing manual, time-consuming documentation.

*“Using tablet is great. It saves time and is much easier than writing on paper. Writing everything down takes long, but with the tablet, I can work quickly. The program has all the information ready, so we just need to click to add it.”* (IDI_23_Clinic assistant)

Participants reported that the digitalized system allowed real-time monitoring which was perceived to facilitate the clinic’s operation and help to maintain the quality of care. Each facility also had a local data server that was linked with the central server for ensuring secure data storage.

*“We have a really smooth data management system here. I can easily monitor patient waiting times, registration duration, consultation time, prescriptions, lab tests, and medicine dispensed. Its a convenient, automated way to track everything.”* (KII_05_Clinic-in-charge)

***Prescription audit*:** Aalo Clinic introduced prescription audits to promote rational prescribing and quality care. With digitalized systems in place, program personnel (senior physicians) could periodically review physicians’ prescriptions and provide feedback which was described as helpful to maintain the quality of care. According to one of the participants, this process was intended to promote the rational use of medicines and raise awareness of inappropriate prescribing practices in Bangladesh.

*“We do prescription audits to ensure drugs are prescribed rationally. In our country, irrational prescribing is common, often aims for quick fixes which is a major reason behind the growing rate of antimicrobial resistance.”* (KII_10_Scheme operator)

***Client feedback system*:** Each clinic had a satisfaction booth where patients could rate the services. This allowed providers to incorporate patient feedback into efforts to improve service delivery. One of the participants reported that,

*“We’ve set up satisfaction booths where patients can rate the services they received. Last month, a few patients expressed dissatisfaction, with the most common reason being long waiting times and occasional medicine unavailability.”* (KII_09_ Scheme operator)

#### Administrative.

***Empanelment*:** Due to the limited number of physicians working in shifts, providing services became challenging when a physician fell sick or took leave. To address this issue, Aalo Clinic initiated an empanelment initiative with the aim of creating a panel of physicians who could step in when any physician was absent. One of the participants perceived that empanelment would allow flexible contracts with physicians, which can make the clinics more efficient. However, another participant reported that during the study period, the empanelment system was not fully operational. One of the participants mentioned that from the initial trial of empanelment, it seemed to be useful in maintaining continuous care.

*“We’ve initiated physician empanelment, a new concept in our context; where one physician can fill in if another one is on leave. Despite challenges, especially in securing physicians for short periods, we’re committed to piloting and refining this approach.”* (KII_4_ Scheme operator)

***Data integration*:** Aalo Clinic’s data was reported to be uploaded into the national health information management system (HMIS), known as the District Health Information Software (DHIS2). The clinic’s digitized system was described as facilitating this process, enabling direct data extraction from the dashboard, ensuring both convenience and data accuracy.

*“Every month, we input data into DHIS2, which is convenient as we can extract data from our dashboard. We also report on maternal healthcare services (ANC, PNC) to the City Corporation, and this information can also be obtained directly from dashboard.”* (KII_01_Clinic-in-charge)

#### Financial.

***Affordable services*:** Aalo Clinic provides consultation services to patients free of charge, along with a limited range of medicines and diagnostic services. Additionally, they refer patients to other facilities with whom they have agreements, where patients can receive services at a discounted price.

*“Whoever comes here can consult a physician for free. We also provide free medicine and limited diagnostic tests, similar to government community clinics. As a PHC center, we don’t have the capacity to provide all lab services, so we refer patients to other facilities with a 25-40% discount using Aalo Clinic’s referral. This helps to reduce out-of-pocket expenses.”* (KII_11_Scheme operator)

### Challenges

#### Technical.

***Limited choice of medicine*:** Patients received free-of-cost medicines at the clinic; however, several providers reported that the available medicines were limited. Frequently, physicians found themselves needing to prescribe medicines that were not included in this list. Physicians reported that the list excluded several high-demand medicines, including those for paediatric, hypertensive, and diabetic conditions. This was perceived to pose a barrier for the low-income population availing healthcare services.

*“Patients often have to buy medicines from outside pharmacies, as we don’t have all the prescribed medicines. It would help if we could add more medicines to our list, like hypertensive and diabetic medications… sometimes they say they can’t afford the medicine, but we have no other option.”* (IDI_11_Physician)

During observation, many clinics were indicated to be experiencing stock-outs of essential medicines. Consequently, the clinic in charge (CIC) could not dispense full dosage of prescribed medications, even those listed as free, leading to frequent partial dispensing and dissatisfaction among beneficiaries.

*“Certainly, it would be better if we could increase the medicine supply. Many patients need calcium, but unfortunately, it’s out of stock. While some patients understand the situation, others don’t.”* (IDI_09_Clinic assistant)

***Hardware barrier*:** Several service providers have reported that their devices (tablets) frequently experience prolonged loading times when accessing patient data or conducting follow-up searches. The sluggish performance of these devices leads to service disruptions, which also found in observation findings.

*“After using the tablets for a while, they start to slow down. This creates long queues, making it challenging for paramedics. Sometimes, the tablet doesn’t respond at all. The problem is getting worse each day.”* (KII_06_Clinic-in-charge)

Some of the participants perceived that the configuration of these devices was not well-suited for their tasks, rendering them prone to slowdowns, especially when dealing with substantial amounts of data.

*“Those Tabs have only two GB RAM. Those devices are not for heavy use. These are for simple tasks.”* (KII_7_Clinic-in-charge)

***Interrupted internet connection*:** The service providers faced challenges with disrupted connections and slow internet bandwidth. Problems with internet connectivity were reported to hamper the service delivery process, as the overall clinic operation relies on digitalization and requires an uninterrupted internet connection. This has led to extended times for consultations and medicine disbursement, resulting in longer queues and waiting times.

*“We often face internet issues. When the connection is disrupted, we get disconnected and have to wait for it to be reloaded. We cannot enter the data or give prescriptions. The patient queue is getting longer … causing us a lot of trouble.”* (IDI_15_Paramedic)

#### Administrative.

***High patient flow*:** As Aalo Clinic’s popularity grew due to its convenient service hours (morning and evening shifts) and affordable services, patient load was reported to increase. According to physicians they usually attended approximately 30–40 patients per shift, and in some instances up to 50 patients. CICs reported turning away a few patients daily due to overwhelming demand. Providers reported that high patient volumes often resulted in waiting times of approximately 15–45 minutes. In some cases, patients were asked to provide their contact information and were given a specific time to return.

*“Health services would improve if the number of physicians was based on the population size. A physician can attend to 30 or 40 patients, sometimes 50 per shift. If there were more patients…. quality can’t be ensured. So, we have to turn down a few patients and ask them to come back the next day.”* (KII_03_Community leader)

Furthermore, this high patient flow has was perceived to create significant pressure on existing resources, increasing the workload of physicians and paramedics. To cope with the high volume of patients, service providers have had to extend their working hours beyond the regular schedule in both shifts.

*“...there is an increased patient load. I can’t leave without attending to all patients, so I ended up staying for extra hours, even during the evening shift.”* (IDI_20_Physician)

***Long waiting time*:** With increased patient flow, the waiting time were reported to increase. The service providers added that at the initial stage, there were limited numbers of patients, and therefore patients did not have to wait for a long time.

*“Sometimes the clinic gets crowded, and patients have to wait for an hour or more…they get disappointed and agitated, but unfortunately, there’s not much we can do. I cannot rush the doctor and compromise the quality of care.”* (KII_05_Clinic-in-charge)

***Referral system*:** Given that the absence of specialized doctors is typical in PHC settings, Aalo Clinic relied on referring patients to higher-level facilities when advanced care was needed. However, there was no established referral system with government facilities. Although referral linkages were initially planned, that could not be implemented due to policy-related barriers. One of the participants perceived that the absence of an enabling government policy hindered the establishment of such linkages.

*“Referral systems are effective only when implemented systematically. Simply providing a referral slip to a patient isn’t enough. We discussed this with policymakers, and they emphasized that a referral mechanism must be clearly outlined in the policy to work.”* (KII_15_Policymaker)

***Space constraints*:** Daily operations were reported to be affected by space limitations. With a notable increase in patient flow, the limited waiting space became a concern, particularly for pregnant and lactating women, and risks breaching patient privacy. Facility observation also indicated that the waiting area was inadequate as patient numbers increased. CICs also claimed difficulties with storage space for medicines, reagents, and equipment.

*“We recently introduced ECG and Ultrasonography, which has made the clinic a bit congested…We need a separate seating arrangement for ultrasonography. Unfortunately, we cannot accommodate these needs within the current setup.”* (KII_12_Scheme operator)

#### Financial.

***Dependency on external funding*:** The model’s sustainability is a key concern due to its reliance on integrating into the government system and the provision of public funding. The necessity for political commitment adds another layer of complexity to this challenge. While a few policymakers expressed concern about the current funding situation, most of them expressed optimism about the model’s potential for future scaling and sustainability.

*“The major challenge I see is sustainability. If the government wants to roll out this nationwide, they need to consider the budget… political commitment will also be crucial. But I don’t think it’s impossible, especially since urban people need this service. Our health sector budget is low, but if we increase it, we can make this happen.”* (KII_17_Policymaker)

## Discussion

This paper offers insights into the implementation of a PHC model, filling a notable gap in research from LMICs, where such evidence is scarce. It provides valuable guidance for decision-making regarding the implementation or refinement of PHC models [22]. While Aalo Clinic presents a promising PHC model for improving access to healthcare services in urban areas of Bangladesh, it faces its own set of challenges. The findings highlight both opportunities presented by the model and implementation-related challenges encountered in its operation, focusing on technical, administrative, and financial aspects.

Aalo Clinic has introduced several innovative approaches within the PHC model including a digitalized system, prescription audit, client feedback system, and empanelment which come with their own unique features and potential benefits. Leveraging technology through the IDHP is a notable strength, facilitating operational efficiency and reducing patients’ hassle of record keeping. Moreover, the paperless digital record system reported to save time, storage, and ensures longer retention of patient records. Studies have shown that electronic health records improve communication between healthcare providers, leading to informed treatment decisions and improved patient outcomes [23]. Furthermore, the use of a unique ID could be scaled up nationwide by incorporating a NID (National Identification) number or a similar identifier ensuring seamless access to patient information and facilitating referrals. These efforts align with broader healthcare IT adoption trends [24], highlighting the Aalo Clinic model as a foundation for expanding technology-driven solutions in Bangladesh. However, challenges with the digitalized systems, such as low-configured devices and interrupted internet, reported to hinder operations, slowed service delivery, and lengthened queues. Similar challenges have been documented on health information systems in LMICs, highlighting the need to upgrade infrastructure and sustainable, locally tailored solutions for successful technology implementation [25]. Notably, Aalo Clinic has transitioned from IDHP to Open Medical Record System (OpenMRS) to align with the national health database (beyond the study period). This transition was reported to enable real-time data transfer, addressing urban health data limitations, while enhancing its scope and ensuring high-quality data for informed decision-making.

Efforts to improve service quality through prescription audits and client feedback systems were described by providers as mechanisms to support adherence to clinical standards and responsiveness to patient concerns. The prescription audit aims to optimize patient care by systematically assessing care against established standards, leading to improved prescription quality and better health outcomes [26]. Additionally, gathering feedback from patients about their healthcare experiences is an essential tool for enhancing the quality of health services provided [27]. A survey reported that 91% of Aalo Clinic users rated their experience positively, indicating a high level of user satisfaction [28]. Aalo Clinic has also adopted anempanelment approach, a fundamental strategy for building or enhancing PHC systems and a key pathway toward achieving UHC [29]. The model empanelled healthcare workers with an aim to ensure continuous access to affordable PHC services through flexible contracts, allowing clinics to adjust staff levels based on the volume of work, thereby enhancing efficiency. Although recruiting adequate physicians for empanelment was challenging, offering attractive packages could incentivize greater participation. Lessons can be learned from models who successfully empanelled physicians such as Delhi’s Mohalla clinic [30]. While effective empanelment is crucial for transitioning towards patient-centred integrated healthcare, there exists limited international guidance on implementing empanelment systems in LMICs [29].

Along with Aalo Clinic’s gained popularity, participants reported increased patient flow, which they described as contributing to longer waiting times and space constraints, consistent with prior research findings [31,32]. Longer waiting times can significantly diminish patients’ satisfaction and willingness to revisit the clinic [32]. Notably despite increased patient flow, the average consultation was reported about 13 minute per patient as reported in a survey [28], which is longer than consultation time reported in many low-and even high-income countries [33]. Additionally, participants reported that space constraints could compromise patient privacy, which could be mitigated through use of screens or temporary partitions. In rural areas, there are currently 13,948 community clinics, each providing PHC to approximately 6,000 people [34]. In contrast, urban areas have only 35 GOD under the MoHFW providing outpatient services, with 19 of them located in Dhaka [34]. Estimates from a quantitative analysis suggested that with one Aalo Clinic per ward in Dhaka city (129 wards total), each clinic would serve 79,680 people. If more clinics are added, three clinics for every ward, the population load per clinic would decrease to 26,560 people [28]. On the other hand, in pluralistic health systems like Bangladesh, an efficient referral system is crucial, providing a systematic approach to ensure appropriate, timely, and affordable healthcare access for the population [35]. However, establishing a referral linkage with government facilities and the Aalo Clinic has proven challenging due to the lack of policies in place. While Aalo Clinic has partnered with a few private facilities for discounted diagnostic tests, these services remain unaffordable for most low-income patients, despite the reduced charges. Financial constraints stand out as a major reason for slum dwellers’ reluctance to seek healthcare [36,37], with the majority belonging to impoverished households, their healthcare choices are significantly influenced by resource availability [9]. Offering free consultations, medicines, and diagnostics at facilities like Aalo Clinic could serve as a pivotal incentive for enhancing healthcare utilization, particularly among the impoverished. However, providers at Aalo Clinic have cited stock-outs of medicines and a demand for a wider range of medications, highlighting the necessity for a need-based supply system.

Aalo Clinic offers several unique features not found in other existing PHC models of Bangladesh, such as GOD [38] or primary healthcare centers (PHCC) under UPHCSDP [39]. These include evening hours services, electronic medical records, and discounted services outside the clinic. While PHCCs offer free registration, medicines, and limited diagnostic tests, these benefits are only available to their ‘red card holders’ (ultra-poor population groups) [39]. Notably, the UPHCSDP is also facing challenges similar to Aalo Clinic [39]. To ensure efficiency in resource use and effective coverage for UHC, existing models should learn from each other. Implementers need to identify ways to integrate popular features of Aalo Clinic into the current PHC models, given their similar structures. Additionally, PHC services could be delivered in collaboration with other private stakeholders, such as NGOs, utilizing the existing facilities of Aalo Clinic. Additionally, domiciliary care by community health workers can be integrated to enhance awareness and promote preventive care in urban areas.

Policymakers have also recognized Aalo Clinic’s potential in providing PHC services for urban residents and emphasized the importance of coordination for integrated healthcare delivery. Moreover, they have underlined the prospect of incorporating Aalo Clinic’s services or features into urban PHC provision under the 5^th^ HNPSP (Health, Nutrition, and Population Sector Program). Such integration would be crucial for the program’s sustainability, leading to more efficient healthcare delivery and benefiting a larger portion of the population in need. The National Urban Health Strategy 2020 acknowledges the existing gaps in urban PHC services and emphasizes the need to enhance capacity, governance, and stewardship of both public and private sectors to ensure equitable and high-quality healthcare for urban populations [40]. This study aligns with the strategy’s objective of generating robust evidence for informed decision-making. However, one of the critical challenges identified in this model is its dependence on external funding, reflecting concerns echoed throughout healthcare financing literature [41,42]. This reliance weakens long-term sustainability, particularly when coupled with the uncertain nature of continued donor support [42]. The requirement for unwavering political commitment adds another layer of complexity [43]. Nonetheless, Aalo Clinic has attracted significant political interest, paving the way for integrating its features into the broader health sector program, which is noteworthy. Further research is required to investigate adaptation strategies for long-term financial sustainability of such models, including alternative funding mechanisms like public-private partnerships or innovative financing models. While considerable progress has been made in PHC reforms across LMICs, this study provides additional implementation insights by examining the Aalo Clinic model in Bangladesh. In line with global PHC reform recommendations, the opportunities and challenges identified in the Aalo Clinic model highlight the importance of integrated service delivery, public-private partnerships, and pro-poor targeting to enhance access and equity [44].

### Strength and limitation

The key strength of this study lies in the fact that the research was designed alongside the implementation of the model which allowed real-time independent observation of the implementation process and informed feasibility and identified modifications required for further refinement of the model. One of the limitations of the study is that observations were conducted over a limited number of visits per site, which may have constrained capturing the full range of implementation variability. Additionally, as it was pilot in nature and therefore the findings are only suitable to inform future designs and scale-up and are not generalizable across different settings.

## Conclusion

Implementing an urban PHC model in a resource-poor setting presents both opportunities and challenges that must be carefully explored to ensure accessible, affordable healthcare services for urban population. The study highlights the importance of addressing implementation challenges while capitalizing on opportunities to scale up the Aalo Clinic model, which has the potential to facilitate the achievement of UHC sin Bangladesh by enhancing service coverage and reducing OOPE. Aalo Clinic offers several innovative features that can be integrated into the existing urban PHC models. Recommendations include implementing structured monitoring, ensuring need-based medicine supply, fostering collaboration with the government for referrals, and facilitating cross-learning among urban PHC facilities. By addressing implementation challenges, leveraging opportunities, and strengthening multi-sector collaboration, the Aalo Clinic model shows potential to contribute to improved access to quality PHC in urban settings, in line with the broader vision of health for all.

## Supporting information

S1 Appendix Interview guidelines. (DOCX)
