# Supplementary material for: Opportunities and challenges of implementing an urban primary healthcare delivery model: Programmatic lessons from Aalo Clinic, Bangladesh
Source: PLoS One. 2026 Feb 9;21(2):e0341924. doi: 10.1371/journal.pone.0341924 (PMC12885247; doi:10.1371/journal.pone.0341924)
Supplement: S1 Appendix — (DOCX) [file pone.0341924.s001.docx]

1. **In-depth Interview (IDI) Guidelines**

**Guideline 1.1: IDI guideline for service providers (GP/ paramedic/ nurse)**

*[****Instructions to the interviewer****: After exchanging greetings; introduce yourself and explain the purpose of your interview to the participant and obtain his/her informed consent using the approved consent form before starting the interview or asking a question.]*

**General Information:**

Name and designation of the participant:

Place of work:

Date of interview:

Interviewer’s name:

**Guidelines:**

1. Background information: Age, gender, education, designation, experience.
2. Did you receive any training on healthcare services for the Aalo Clinic? ***(Probe:*** *antenatal care, postnatal care*, *newborn care, child immunization, breastfeeding, nutrition, adolescent reproductive health, counseling, etc.*). If not, what kind of training do you need? Specify, please.
3. Do you have any treatment protocol to provide services to the people? Is this sufficient to provide care? (***Probe:*** *different services provided at the Aalo Clinic*)? Would you please tell me the service delivery process in your facility? (***Probe:*** *from the patient come to the hospital and go back home).*
4. Please share your experience with the supply of drugs, availability, and their provision to the patients. Please tell us about the diagnostic test services provided in the Aalo Clinics, and the availability of medical equipment (X-ray, USG, ECG, equipment, oxygen, nebulizer, and other emergency and basic supplies)
5. Please tell us about the referral system of Aalo Clinic. (***Probe:*** *within the Aalo clinic, outside Aalo clinic, follow up for referral patients*). Does your organization have a partnership with any organizations for the referral? What are the challenges regarding the referral? What will be the possible solutions/suggestions? What should be done to ensure sustainability in the referral system?
6. What do you think about the current MIS? Effectiveness of MIS regarding record-keeping and reporting? What should be done to bring it into regular practice? (***Probe:*** *need of supporting staff, system development, user friendly, etc.*). In your opinion, how to make it user-friendly?
7. What challenges do you face in Human Resources needed for Aalo Clinic’s pilot? How do you manage the shortage of workforce?
8. Do you have a monitoring system in your work? What challenges do you face in monitoring and supervising of Aalo Clinic model to provide quality care?
9. What is your opinion about the satisfaction booth? Do people drop their opinions in this booth with proper privacy? Were there any initiatives based on the patient’s feedback? How does the Aalo Clinic authority deal with them?
10. What is your observation/ suggestion on the IT-based record-keeping system (automation) of the Aalo Clinic model?
11. From what source do people get information about healthcare? (***Probe:*** *posters, stickers, billboards, radio programs, television, etc.)*. Do you think this information is sufficient? If not, what can be the possible way?
12. What are the main challenges to providing services for the patients? What are the major gaps to ensure Aalo Clinic’s sustainability? How can you deal with these?
13. Do you think Aalo Clinic is tested enough to go for the future scale-up? What would you suggest before scale-up?

**Guideline 1.2: IDI guideline for service providers (lab** **technician/lab manager/clinic assistant)**

*[****Instructions to the interviewer****: After exchanging greetings; introduce yourself and explain the purpose of your interview to the participant and obtain his/her informed consent using the approved consent form before starting the interview or asking a question.]*

**General Information:**

Date of interview:

Interviewer’s name:

**Guidelines:**

1. Background information. (***Probe:*** name, age, gender, education, occupation, etc.).
2. As a contract-based organization, how did you get involved in the activities of the Aalo Clinic model?
3. Tell us about your roles and responsibilities in the service delivery model. What are the main challenges in your responsibilities (***Probe:*** *working hours, shifting duty, etc.*)? How do you deal with them?
4. What kind of medicines/tests are provided? Do you have all the medicines/ test equipment? What are the difficulties you faced in providing services (***Probe:*** *lack of co-operation, workload, patient flow, etc.*)? How do you deal with them? Please tell me the details.
5. Please explain the professional qualifications, training, and skills required for the Aalo Clinic pharmacist/ lab technologist. (***Probe:*** *educational qualifications, training, etc.)*
6. What measures do you take to maintain and preserve the quality of medicines/ test kits?
7. What kind of initiative has been taken for better medicine with lower cost? Do you have any challenges with the inventory management system (***Probe:*** *setup, safekeeping, supply, etc.*? How do you deal with them?
8. What other benefits do you think can be added to the model to get better results?
9. What do you think should be the future strategy for the sustainability of this model?
10. **Key Informant Interview (KII) Guidelines**

**Guideline 2.1: KII guideline for policymakers**

*[****Instructions to the interviewer****: After exchanging greetings; introduce yourself and explain the purpose of your interview to the participant and obtain his/her informed consent using the approved consent form before starting the interview or asking a question.]*

**General Information:**

Name of interviewee and designation:

Organization name:

Date of interview:

Name of interviewer:

**Guidelines:**

1. Please tell us about the urban healthcare system and healthcare service availability in urban areas.
2. What do you think about the current situation of delivering PHC in the urban healthcare system? Please briefly share your role in the urban PHC system.
3. Please tell us about the newly developed Aalo Clinic model.
4. In your opinion, how effective is the referral system under the Aalo Clinic model? Do you see any challenges regarding the referral of patients? What are those? How to minimize these challenges?
5. How are the local government agencies involved with the Aalo Clinic model? What are their roles and how effectively do they function? Would you provide any suggestions for effective engagement?
6. What are the key barriers to implementing the Aalo Clinic model? (***Probe:*** *Human resources, funding, basic amenities, IT-based data management system, etc*.). How those barriers could be addressed?
7. What are the possibilities of scaling up the Aalo Clinic model in other areas? What do you think about the current capacity of the Government of Bangladesh (GoB) in managing the future scale-up? Do you foresee any scale-up-related challenges? How will you minimize those challenges? What could be the possible organizational and legal framework for the future?
8. How to integrate the Aalo Clinic model into the government healthcare system? Will the existing policies allow this? If not, what measurements should be taken?
9. What would you suggest to improve the Aalo Clinic model’s sustainability? Do you see any policy barrier to the sustainability of the model? If so, what are those? How to overcome those barriers?

**Guideline 2.2: KII guideline for community leaders**

*[****Instructions to the interviewer****: After exchanging greetings; introduce yourself and explain the purpose of your interview to the participant and obtain his/her informed consent using the approved consent form before starting the interview or asking a question.]*

**General Information:**

Name and designation of the participant:

Place of work:

Date of interview:

Interviewer’s name:

**Guidelines:**

1. Background information. (***Probe:*** name, age, gender, education, occupation, etc.).
2. Where do you or your community people usually seek healthcare? (***Probe:*** *Pharmacy, Consultation Center, Medical College Hospital, Other Government Hospitals)*.
3. Have you heard about Aalo Clinic? Do the community people know about the Aalo Clinic?
4. Do you know what services are available in the Aalo Clinic? Tell the details. (***Probes*** *Medicine, Diagnostic tests, Referral services, etc.)*
5. How do you inform and encourage your community people to seek primary healthcare from Aalo clinics? What activities do you perform to encourage the community to seek care from the Aalo Clinic in your area? (***Probe:*** counselling, miking, campaigning, monthly meetings, etc.). Please share your experiences.
6. In your opinion, do you see any improvement in the primary healthcare services in your area by Aalo Clinic? What is the possibility of implementing Aalo clinics in the urban area? In other words, what role do you think this new model is playing in improving primary healthcare in urban people?
7. What would you suggest to improve the programmatic sustainability of the Aalo Clinic’s model?

**Guideline 2.3: KII guideline for facility managers**

*[****Instructions to the interviewer****: After exchanging greetings; introduce yourself and explain the purpose of your interview to the participant and obtain his/her informed consent using the approved consent form before starting the interview or asking a question.]*

**General Information:**

Name of interviewee and designation:

Date of interview:

Name of interviewer:

**Guidelines:**

1. Please share your experience of service provision (***Probe***: *available services, types of treatment provided, etc.*) and utilization of the Aalo Clinic model. Would you please tell me the service delivery process in your facility? (***Probe:*** from the patient come to the hospital and go back home).
2. Do you have an adequate supply of drugs, diagnostic tests, and equipment (X-ray, USG, ECG, OT equipment, oxygen, nebulizer, and other emergency and basic supplies)? Please share your experience with the supply of drugs, diagnostic tests, and equipment. Do you face any issues/challenges regarding these? What would you suggest to improve the supply chain management?
3. How do you manage the referral patients and the challenges related to appropriate referrals posed by the Aalo Clinic model? What is your opinion about the effectiveness of the Aalo Clinic referral system?
4. What sorts of challenges do you face regarding the human resource/financial/monitoring-supervision, IT-based data management system to provide quality care? How do/will you overcome these?
5. What is your opinion about the job satisfaction of the service providers (doctor, nurse, paramedic, lab technologist, pharmacist, guard, cleaner, aya, etc.) (***Probe:*** income, working environment, working hours, etc.)?
6. Please tell us about the role of and coordination mechanism among different stakeholders (***Probe:*** scheme operator, contracted diagnostic, contracted pharmacy, etc.). In your opinion, what are the potential challenges for effective coordination, and how to mitigate these?
7. What initiatives are taken to promote the Aalo Clinic? Are there any community engagement activities? Please describe them. What is the source of information to know about the Aalo Clinic and its health care services? (***Probe:*** *campaign, door-to-door visit*, *posters, stickers, billboards, radio programs, television, etc.)*.
8. What is your opinion about the satisfaction booth? Do people drop their opinions in this booth with proper privacy? Were there any initiatives based on the patient’s feedback? How do the Aalo Clinic authorities deal with them?
9. Would you summarize the lessons learned in terms of strengths, weaknesses, opportunities, and challenges of the Aalo Clinic model?
10. In your opinion, what changes (***Probe:*** *ownership, financial management, implementation modality, infrastructure, HMIS system, satisfaction booth (privacy, effectiveness, usage*) are required to improve the programmatic sustainability of the Aalo Clinic model? Please elaborate the issues.

**Guideline 2.4: KII guideline for scheme operator**

*[****Instructions to the interviewer****: After exchanging greetings; introduce yourself and explain the purpose of your interview to the participant and obtain his/her informed consent using the approved consent form before starting the interview or asking a question.]*

**General Information:**

Name of interviewee and designation:

Organization name:

Date of interview:

Name of interviewer:

**Guidelines:**

1. Please tell us about the overall idea of the Aalo Clinic Model.
2. As a contract-based organization, how did you get involved with the Aalo Clinic model? Tell us about your roles and responsibilities in the service delivery model.
3. How to manage the computer-based database? Who manages it? And how is the database updated? Effective use of the IT-based system (***Probe***: *monitoring, service usage, user-friendliness, etc.*).
4. How do you refer the patient? What kind of facility do you provide for the patients (***Probe:*** *referral hospital-related information*? How do you deal with the patients for further follow-up?
5. Tell us about your monitoring and supervision system. (***Probe:*** *who monitors, how monitors*)
6. Tell us about your reporting system. (***Probe:*** *to whom, how to report, how long to report?*)
7. In your opinion, what are the challenges in performing the responsibilities of the model-related activities as a contract-based organization? What steps have been taken to address the challenge? (***Probe:*** *referral, medicine, diagnostic tests services, consultation*).
8. Did your organization take any initiative related to promoting Aalo Clinic? What was the implementation of the community engagement activities? From what source do people get information about receiving health care services? (***Probe:*** *campaign, door-to-door visit*, *posters, stickers, billboards, radio programs, television, etc.)*.
9. How effective is the satisfaction booth? Were there any initiatives based on the patient’s feedback? How do the Aalo Clinic authorities deal with them?
10. What other benefits can be added to the model to get better results?
11. In your opinion, what changes (***Probe:*** *ownership, financial management, implementation modality, infrastructure, HMIS system, satisfaction booth (privacy, effectiveness, usage*) are required to improve the programmatic sustainability of the Aalo Clinic model? Elaborate/segregate the issues.
12. What do you think should be the future strategy for scaling up the Aalo Clinic model and its management of this?
